# Tetrodotoxin: The State-of-the-Art Progress in Characterization, Detection, Biosynthesis, and Transport Enrichment

**DOI:** 10.3390/md22120531

**Published:** 2024-11-26

**Authors:** Xinxin Zhang, Kun Qiao, Ruimin Cui, Min Xu, Shuilin Cai, Qilin Huang, Zhiyu Liu

**Affiliations:** 1 College of Food Science and Technology, Huazhong Agricultural University, Wuhan 430070, China; 18764245595@163.com; 2 Key Laboratory of Cultivation and High-Value Utilization of Marine Organisms in Fujian Province, Fisheries Research Institute of Fujian, National Research and Development Center for Marine Fish Processing, Xiamen 361021, China; qiaokun@xmu.edu.cn (K.Q.); xumin1315@foxmail.com (M.X.); caishuilin@hqu.edu.cn (S.C.); 3 College of Food Science and Technology, Zhejiang Ocean University, Zhoushan 316022, China; cuiruimin@zjou.edu.cn

**Keywords:** tetrodotoxin, puffer fish, TTX biosynthesis, TTX transporter accumulation

## Abstract

Tetrodotoxin (TTX) is a neurotoxin that binds to sodium channels and blocks sodium conduction. Importantly, TTX has been increasingly detected in edible aquatic organisms. Because of this and the lack of specific antidotes, TTX poisoning is now a major threat to public health. However, it is of note that ultra-low dose TTX is an excellent analgesic with great medicinal value. These contradictory effects highlight the need for further research to elucidate the impacts and functional mechanisms of TTX. This review summarizes the latest research progress in relation to TTX sources, analogs, mechanisms of action, detection methods, poisoning symptoms, therapeutic options, biosynthesis pathways, and mechanisms of transport and accumulation in pufferfish. This review also provides a theoretical basis for reducing the poisoning risks associated with TTX and for establishing an effective system for its use and management to ensure the safety of fisheries and human health.

## 1. Introduction

Tetrodotoxin (TTX) is a neurotoxin that can block nerve conduction by binding to sodium channel receptors [1]. TTX was first isolated from pufferfish (*Tetraodontidae*) in 1909, after which it is named. It is characterized as a crystalline, weakly alkaline small-molecule alkaloid with the chemical formula C_11_H_17_O_8_N_3_, a molecular weight of 319, solubility in acidic solutions [2], and thermal stability. Crucially, TTX can be highly toxic, with an LD_50_ of 8.7 μg/kg, which is 1200 times higher than that of cyanide, and its toxicity is not compromised by conventional cooking heat [3,4]. Specifically, <10 MU/g (i.e., 2.2 mg/kg; MU: mouse unit, 1 MU [5] indicates the average amount of toxin that is lethal to mice weighing 20 g within 30 min.) of TTX is considered non-toxic, but >1.5–2.0 mg of TTX (equivalent to a blood level of 9 ng/mL) is lethal for humans [6].

To date, the origin of TTX is not fully understood. Numerous studies have shown that TTX is produced by specific bacteria (*Vibrio* spp. and *Bacillus* spp., among others) [7], which may be either symbiotic with organisms accumulating TTX or enriched in the organisms through the food chain. Melnikova et al. [8] isolated the TTX-producing bacterium *Bacillus* sp. 1839 from a marine banded worm and sequenced the complete genome of the bacterium, which may play a crucial role in the biosynthesis of TTX in marine ecosystems. Secondly, TTX-carrying organisms in the ocean, especially pufferfish, bivalves, and gastropods, may acquire TTX by consuming TTX-containing foods (microalgae and flatworms, etc.) [8,9,10]. However, some studies have suggested that certain species, such as newts [11], may have the ability to synthesize TTX endogenously, raising questions about the evolutionary pathways of TTX production in terrestrial and aquatic environments. The sources of TTX are currently thought to be multifaceted, involving bacterial production, dietary accumulation, and potential endogenous synthesis by certain organisms. The interactions between these factors, as well as environmental influences, lead to the complex dynamics of TTX in ecosystems.

The biosynthesis of TTX has attracted the interest of several scholars due to its complex structure. TTX biosynthesis in salamanders has been reported to be derived from monoterpenes [12]. Based on the complete genome of TTX-producing Bacillus subtilis, the ability to produce bacterial TTX biosynthesis may be associated with specific gene clusters related to polyketide synthase (PKS) and non-ribosomal peptide synthetase (NRPS), which are usually involved in the biosynthesis of complex natural products [8]. In addition to bacterial and salamander sources, TTX biosynthesis may also occur in marine organisms such as river herrings, where TTX is synthesized as a series of enzymatic transformations of TTX precursors to unique TTX structures [13,14].

Studies on the mechanism of TTX transport and accumulation in organisms, especially in pufferfish, have been reported in recent years. However, the mechanism of TTX transport and its accumulation among various tissues of pufferfish has not yet been clarified. Why is pufferfish resistant to highly toxic substances? Research suggests that there are two primary mechanisms by which pufferfish can resist highly toxic substances. The first mechanism involves a mutation in the sodium channel that attenuates or inhibits the binding of TTX to the sodium channel. The substitution of non-aromatic amino acid chains for aromatic amino acid chains in the pore ring of structural domain I of Na_V_1.4 in pufferfish effectively reduces their binding to TTX [15,16]. The second is the production of TTX-binding proteins. TTX-binding proteins effectively transport TTX to specific organs. Therefore, TTX-binding proteins play an important role in the transport and accumulation of TTX in pufferfish, and the saxitoxin- and tetrodotoxin-binding protein (PSTBP) has been found in their plasma, which promotes the transport of TTX between tissues, especially to the skin, which is capable of secreting TTX [17]. Exploring the regulatory mechanism of TTX-binding proteins is the key to studying the mechanism of TTX transport accumulation in organisms.

Pufferfish have historically been a part of the cultural diets of China, Japan, and other Asian countries because their meat is considered desirable; however, the high toxicity of pufferfish is an ongoing concern. There are often incidents of poisoning caused by improper handling or accidental consumption of highly toxic pufferfish [18,19,20,21,22,23,24]. Therefore, the potential threat of TTX to human health and safety has become a global concern. In 1941, Fukuda and Tani [25] categorized the symptoms of TTX poisoning into four grades, of which grades 3 and 4 would have life-threatening symptoms such as respiratory failure and cardiac arrhythmia. The clinical treatment of TTX is to promote the body’s elimination of TTX because there is no effective antidote. Since there is no specific antidote for TTX, clinical treatment is based on inducing vomiting, as well as respiratory and cardiac function assistance to facilitate the body’s elimination of TTX. Although TTX is a highly toxic substance, due to its selective sodium channel blocking property, at low doses, it can be used as a palliative agent in the later stages of certain cancers to treat neuropathic and visceral pain [26]. The remarkable efficacy of TTX in treating cancer pain was confirmed by clinical tests. Hagen et al. [27] studied the analgesic effects of TTX for the treatment of pain in patients with cancer and showed that the average pain response in the TTX injection group was significantly lower than that in the placebo group, indicating that TTX is an effective analgesic for patients with moderate-to-severe cancer. Halneuron^TM^ Wex Pharmaceutical Inc. (Vancouver, BC, Canada) is developing TTX as a pain reliever for patients with mid-to-late-stage cancer, and phase III clinical trials were conducted in 2019. Thus, TTX is expected to become a routine clinical treatment in the near future [28]. Another advantage of TTX is that it does not produce side effects such as addiction, drowsiness, respiratory depression, or cognitive decline, which are common with opioids [26,29]. Furthermore, TTX is particularly suitable for treating neuropathic pain. Despite its enormous potential pharmaceutical value, TTX is currently primarily extracted from TTX-containing biological organs, resulting in low yields and serious wastage. Thus, there is a clear need to elucidate the biosynthetic processes involved in TTX generation to increase its yield and realize its full potential as a therapeutic drug. However, the biosynthetic process of TTX and the transcriptional enrichment mechanism in pufferfish are rarely discussed.

The aim of this review is to summarize the latest research progress regarding TTX sources, analogs, mechanisms of action, biosynthesis pathways, mechanisms of transport and accumulation in pufferfish, detection methods, poisoning symptoms, and therapeutic options. This review provides a theoretical basis for reducing the poisoning risks associated with TTX and for establishing an effective system for its use and management to ensure the safety of fisheries and human health.

## 2. Tetrodotoxin Sources

More than 150 species of TTX-producing bacteria have been identified, indicating that bacteria are the primary producers of TTX in nature. However, the conclusions of existing studies on the origin of TTX in aquatic organisms are still controversial, and there are two main hypotheses: exogenous food chain accumulation and endogenous bacterial symbiosis. For the endogenous bacterial symbiosis hypothesis, in 1986, Noguchi et al. [30] (1986) first isolated *Vibrio* spp. from the intestine of the crab *Atergatis floridus* and detected the presence of TTX and its dehydrated analogs in cell extracts and culture media using HPLC-FLD and GC-MS methods. More TTX-producing genera were subsequently discovered, e.g., *Pseudomonas* spp. isolated from the red algae *Jania* sp. [31], *Vibrio alginolyticus* isolated from the intestine of *Fugu vermicularis* [32], and *Bacillus* spp. and *Actinobacteria* spp. identified in the ovaries, liver, and intestines of other pufferfish [33]. It has been suggested that TTX is produced by commensal microorganisms in the carrying organisms. However, this theory is accompanied by some doubt, as the limited number of commensal microorganisms makes it difficult to produce the same levels of TTX found in TTX carriers. In contrast, the hypothesis of exogenous food-chain accumulation has been accepted by the majority of the population. Noguchi et al. [34] tested this hypothesis experimentally by keeping TTX-carrying organisms in isolation for 1 year and found that more than 5000 samples of pufferfish kept in nets or terrestrial aquariums became non-toxic. When the non-toxic pufferfish were fed food containing TTX, the levels of TTX in their bodies increased, further supporting the idea that TTX accumulates through the food chain.

Newts (*Cynops*) are representative amphibian organisms that carry TTX, and their skin and eggs are the primary tissues in which TTX accumulates. Studies have found extremely high concentrations of TTX in the skin of highly poisonous newts, enough to pose a lethal threat to any potential predators [35]. There is also some controversy as to whether newts produce TTX endogenously or accumulate TTX through the food chain, and the recent discovery of TTX in fly larvae *Limnophilus* spp., as well as terrestrial flatworms *Bipalium adventitium*, and *Bipalium kewense*, provides new theoretical support for the salamander TTX exogenous hypothesis [36,37]. In addition, Yotsu-Yamashita et al. [38] conducted non-toxic feeding experiments on newts (*Notophthalmus viridescens*) for 3 and 6 years, and the results showed a significant decrease in TTX content in newts, and no TTX was detected in newts that had been kept in captivity for 6 years. These findings provide strong evidence for the idea that amphibians are enriched in TTX through exogenous food. However, some studies have also found that TTX carried by newts is endogenous; the skin glands of captive poisonous newts can regenerate and secrete TTX [39]. Gall et al. [40] reported that TTX was identified in eggs laid by long-term captive female newts *Taricha granulosa*. In addition, studies of the body surface and internal microbes of newts have shown that no TTX-producing commensal bacteria are present in or on their skin surface [41]. Mailho-Fontana et al. [42] found differences in the proportions of type 1 and type II secretory cells in the skin glands of toxic and non-toxic newts, with a larger and higher proportion of type 1 secretory cells in highly toxic newts, which may be related to TTX levels. All the above studies indicate that TTX, carried by newts, has endogenous possibilities.

## 3. Tetrodotoxin Analogs

**Figure 1:**
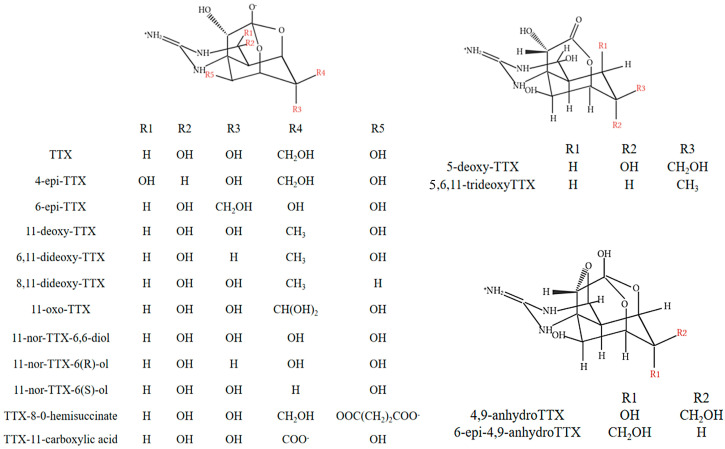
The structure of TTX and its analogs.

At least 30 TTX analogs have been reported and classified based on their structures as hemilactal, lactone, and 4,9-anhydrous types. The structures of TTX and its analogs are shown in Figure 1. Bane et al. [43] mentioned 26 naturally occurring TTX analogs, of which 5,6,11-trideoxyTTX is the most predominant analog in pufferfish and gastropod tissues [44,45]. Noguchi et al. [46] showed that pufferfish (*Takifugu niphobles*) could specifically perceive and are sensitive to the odor of 5,6,11-trideoxyTTX, suggesting that this is a chemical attractant for pufferfish. The results were confirmed by several other studies [47,48]. In addition, some new TTX analogs have been discovered in recent years. Kudo et al. [49] discovered new N-hydroxy TTXs in their study of the TTX anabolic pathway in newts: 8-epiTTX, 1-hydroxy-8-epiTTX, 1-hydroxy-8-epi-511-dideoxyTTX, and 4,9-anhydro-10-hemiketal-5-deoxyTTX. Tonon et al. [50] isolated four novel TTX analogs, methyl-TTX, TTX-acetate, hydroxypropyl-TTX, and glycerol-TTX, in the tissues and symbiotic microorganisms of two pufferfish species (*Sphoeroides spengleri*, *Canthigaster figuereidoi*); and 9-epiTTX, which was found in the river puffer *Takifugu flavipterus* [10]; and 9-epiTTX found in the pufferfish *Takifugu flavipterus*.

The toxicity of TTX analogs varies depending on the chemical structure. Since there are no certified standards for TTX analogs, the strength of their toxicity is currently assessed based on toxicity equivalency factors [51] (TEFs: the toxicity ratio of a compound from a chemical group that shares the same mode of action of a reference compound in the same group). It was found that 11-oxoTTX and 5,11-dideoxyTTX were relatively more toxic with TEF = 0.75, and other known analogs had low-toxicity TEFs (range = 0.2–0.011). Alkassar et al. [52] assessed the TEFs of TTX analogs using an assay with Neuro-2a cells and found that several analogs, 5,11-dideoxyTTX, 11-norTTX-6(S)-ol, 11-deoxyTTX, and 5,6,11-trideoxyTTX, were less toxic than TTX (TEFs = 0.75–0.011), with 11-norTTX-6(S)-ol having a TEF of 0.404, whereas Botana et al. [53] found that 11-norTTX-6(S)-ol had a TEF = 0.185 using a mouse bioassay. In addition, Reverté et al. [54] determined the TEFs of five TTX analogs using an automated patch-clamp technique. This study represents the first application of this method to assess the toxicity of TTX analogs. Although there were significant discrepancies compared to previously reported TEF values, the findings of their assay aligned with the results obtained from the mouse bioassay, cellular assays, and other methodologies. Specifically, the toxicity of the five TTX analogs tested was found to be lower than that of TTX itself. Furthermore, since the obtained TTX analogs in the laboratory were obtained using bioseparation techniques, it is hypothesized that the purity and source of the analogs caused the difference in TEF results, as shown in Table 1.

## 4. Tetrodotoxin and Voltage-Gated Sodium Channels

### 4.1. TTX Mechanism of Action

**Figure 2:**
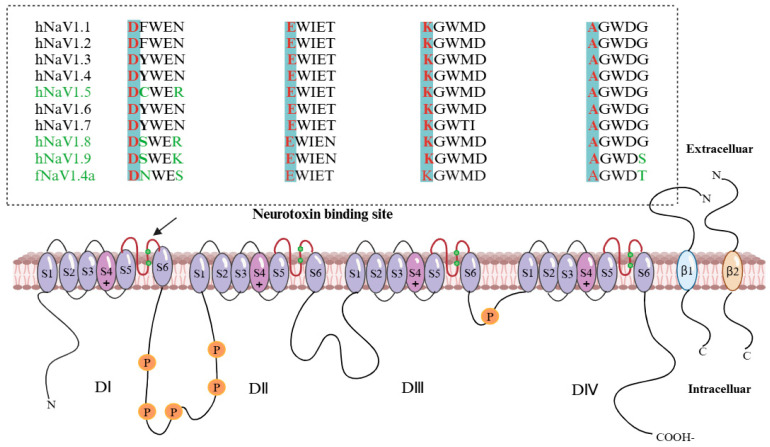
Amino acid sequences of the binding sites (*α*-subunit) for human and pufferfish sodium channels to TTX [15,70,71]. DI, DII, DIII, DIV: four homologous protein structural domains of the sodium channel *α*-subunit; S1–S6: six hydrophobic transmembrane segments of each homologous protein structural domains. Between S5 and S6 is the TTX binding site (green dot); DEKA: Amino acid sequences, Green color represents different amino acids.

The directed flow of Na^+^ generated by voltage-gated sodium channels (Na_V_) underlies the initiation and propagation of action potentials in nerve and muscle fibers. TTX is a sodium channel blocker that selectively inhibits certain sodium channel alpha subunits. The sodium channel α-subunit is a voltage-sensing structure consisting of four homologous protein structural domains, each containing six hydrophobic transmembrane segments (S1–S6) that form an ion-selective filter on the outside of the cell [60,61]. The ion-selective filter contains two highly conserved amino acid sequences, DEKA and EEMA (EEID in invertebrates), which are TTX binding sites. The TTX can also bind to aromatic amino acid residues downstream of aspartic acid (D) in the DEKA sequence. Furthermore, TTX can also bind directly to aspartic acid (D) in the EEMD loop [62,63,64,65], which clogs the sodium channel pores and prevents the normal flow of Na (see Figure 2). Six of the previously identified mammalian sodium channels exhibit sensitivity to TTX, namely Na_V_1.1, Na_V_1.2, and Na_V_1.3, which are highly expressed in the CNS (TTX IC_50_ 5.9, 7.8, and 2.0 nM, respectively), Na_V_1.4, the major sodium channel in skeletal muscle (TTX IC_50_ = 4.5 nM), and TTX in neurons of the CNS and the peripheral nervous system. Na_V_1.6 (TTX IC_50_ = 3.8 nM) is expressed in neurons of the peripheral nervous system, and Na_V_1.7 (TTX IC_50_ = 5.5 nM) is also found in the peripheral nervous system. In addition, three Navs were found to be much less sensitive to TTX: Na_V_1.5 in the heart showed antagonistic effects on TTX (TTX IC_50_ = 1.97 μM) and the Na_V_1.8 and Na_V_1.9 expressed in dorsal root ganglion neurons were also insensitive to TTX (TTX IC_50_ 1.33, 5.96 μM, respectively) [66,67,68,69].

### 4.2. Pharmacological Value of Tetrodotoxin

Mutations in sodium channels are associated with a wide range of diseases. To date, hundreds of mutation sites in the *α-* and *β*-subunits of the sodium channels have been identified in relation to 50 human disorders, including epilepsy, neuropathic pain, and cardiac arrhythmias [72,73]. Elucidating the role of sodium channels in health and disease could aid in drug development and disease treatment. Mutations in several Na_V_-*α* subunits were associated with epilepsy, including Na_V_1.1 (*SCN1A*), Na_V_1.2 (*SCN2A*), Na_V_1.3 (*SCN3A*), Na_V_1.6 (*SCN8A*), and Na_V_1.7 (*SCN9A*) [74]. Of these, Na_V_1.1, Na_V_1.2, and Na_V_1.6 are the major sodium channels in the brain, accounting for approximately 95% of the total sodium channels. Meisler et al. [75] conducted knockout experiments for *SCN1A*, *SCN2A*, and *SCN8A* in mice. Animals with a knockout of all three genes died: the loss of function of *Scn1A* (Na_V_1.1) resulted in seizures; the loss of function of *Scn2A* (Na_V_1.2) resulted in respiratory failure and death in neonatal mice; and the loss of function of *Scn8A* (Na_V_1.6) resulted in the failure of neuromuscular junctions and hind limb paralysis, culminating in death at 3 weeks of age. In addition, Na_V_1.3 mutations have recently been found to be associated with congenital epilepsy; Na_V_1.3 is also associated with pain transmission, and peripheral neuronal injury leads to Na_V_1.3 overexpression in the hypothalamic neurons and dorsal root ganglia [74,76]. Studies that investigated neuropathic pain disorders showed that Na_V_1.7, Na_V_1.8, and Na_V_1.9 play important roles in the peripheral signaling of pain [77]. Specifically, loss-of-function mutations in *SCN9A* can cause the congenital absence of pain (CIP) in humans [78], which is associated with acute trauma (burns, bumps) as well as persistent pain from trauma and disease [64]. Mutations in Na_V_1.5, which are found primarily in the heart, cause tardive dyskinesia/Brugada syndrome [79,80], which is a condition associated with sudden cardiac death in young adults. Barc et al. [81] found mutations in the *SCN5A* and *SCN10A* susceptibility genes, indicating that the function of sodium channels is associated with patients at risk for Brugada syndrome. Today, drugs on the market for pain relief and antiepileptic drugs work primarily by blocking sodium channels [82]. Thus, there is great potential for the treatment of diseases associated with sodium mutations and substances that can act on sodium channels.

TTX is a potent sodium channel blocker that is less sensitive in the cardiac major Na_V_1.5. Consequently, TTX has great development potential as a novel therapeutic drug and anesthetic. Clinical studies have demonstrated that even at doses well below the LD_50_ dose, TTX significantly reduces the pain caused by different conditions, with particular excellence in the treatment of mid-to-late-stage cancer pain, neuromuscular pain, and visceral pain. In addition to studying the effects and applied doses of TTX on various diseases in clinical trials and animals, strengthening the efficacy of TTX and reducing the therapeutic dose is another hot research topic. Epinephrine, bupivacaine, and chemical osmotic enhancers can improve the efficacy of TTX and reduce its side effects, which include abnormal sensations in the face or mouth, headache, dizziness, nausea, and myalgia. Kohane et al. [83] showed that epinephrine can reduce the action of TTX concentration from 37.6 μM to 11.5 μM and prolong its nerve block; bupivacaine enhanced the local anesthetic effect of TTX and reduced systemic toxicity. In addition, Santamaria et al. [84] found that chemical osmotic enhancers (sodium octyl sulfate and octyl trimethyl ammonium bromide) prolonged the duration of the TTX nerve block but did not reduce systemic toxicity.

**Figure 3:**
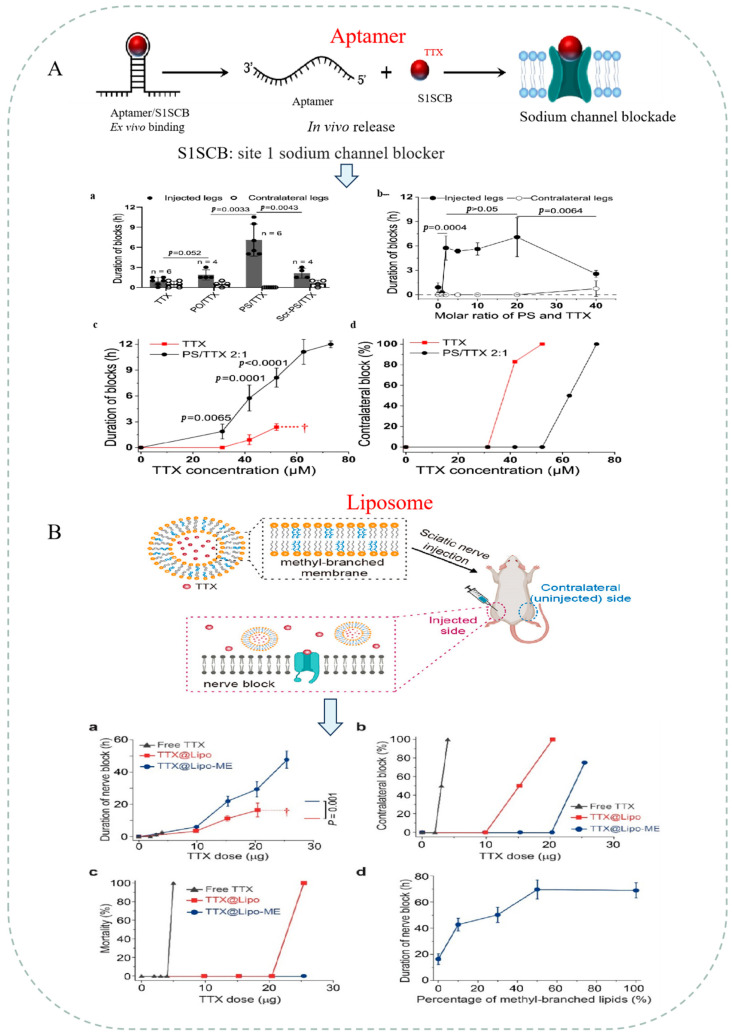

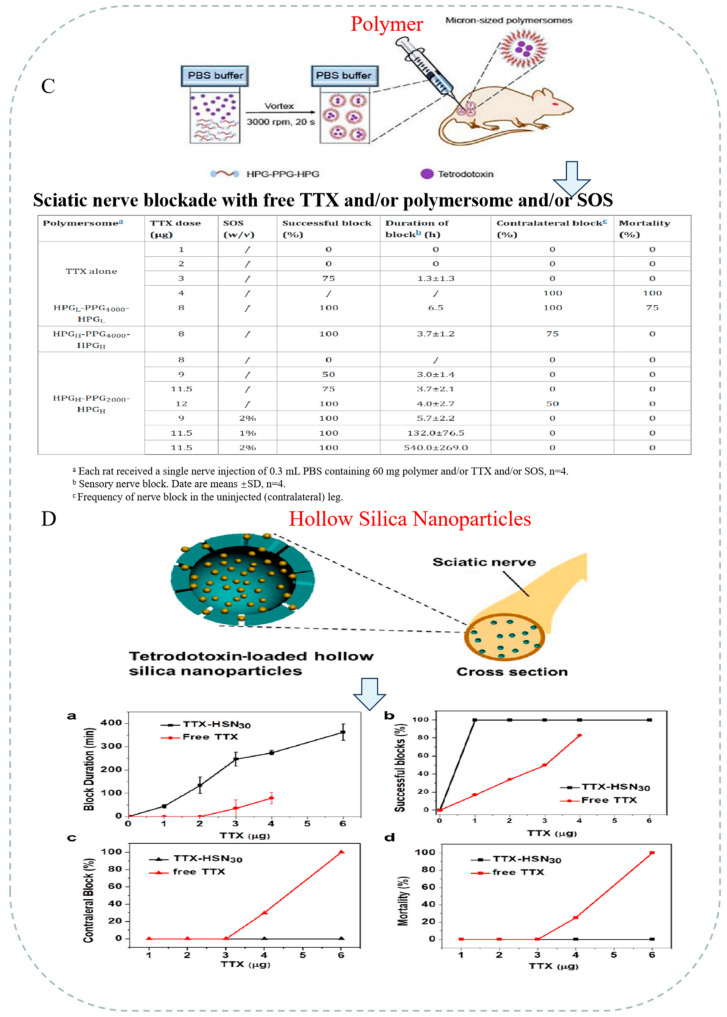
Enhanced TTX nerve block effect and targeted anesthesia. (**A**): Schematic illustration of aptamer/TTX complexes and the block time on the rat sciatic nerve [86]; a. Peripheral nerve blockade with 42 µM TTX, free or complexed with aptamers (PO, PS, Scr-PS); b. Peripheral nerve blockade with varying molar ratios of PS/TTX complexes, using 42 µM TTX; c. Sciatic nerve blockade with free TTX and PS/TTX (2:1) in the injected hindpaws. The dagger indicates 100% mortality; d. Frequency of nerve block in the contralateral (uninjected) hindpaws; (**B**): schematic illustration of liposome-encapsulated TTX and the block time on the rat sciatic nerve [87]; a. Duration of sensory nerve block from different formulations. Daggers indicate 100% mortality; b. Frequency of block in the contralateral (uninjected) leg; c. Mortality from different formulations; d. Sensory nerve block with different molar percentages of branched lipids in the liposomal formulation, using 25 μg TTX; (**C**): schematic illustration of polymer and TTX system, and the block time on the rat sciatic nerve [88]; and (**D**): schematic illustration of hollow silica nanoparticles capped with TTX, and the block time on the rat sciatic nerve [89]; Sciatic nerve blockade with free TTX and TTX-HSN30. Effect of TTX dose on a. the median duration of sensory nerve blocks, b. the frequency of successful blocks, c. the frequency of nerve block in the uninjected (contralateral) extremity, and d. of death.

In addition to the above substances, thiophosphate-modified aptamers, liposomes, and inorganic nanoparticles encapsulating TTX have shown good neuroblocking effects in clinical applications or pilot studies, prolonging the in vivo release of TTX and reducing systemic toxicity [85,86]. Wang et al. [86] utilized phosphorothioate-modified aptamers to specifically bind to TTX to form non-covalent aptamer/drug complexes that significantly prolonged the drug administration time and reduced systemic toxicity. The high specificity and modifiability of the aptamer with TTX provide great potential for its use as a delivery vehicle for TTX. Li et al. [87] modified liposomes with methyl-branched phospholipids, resulting in a 10–14% increase in the sealing capacity of the liposomes for TTX and extending anesthesia duration up to 70 h. The aptamer has also been identified as a promising candidate for TTX drug delivery. Similarly, Li et al. [88] used micron-sized emulsion-induced polymers to encapsulate TTX, and the results showed that the TTX encapsulation efficiency reached 94%, which resulted in stable release and prolonged analgesia; by injecting an emulsion-induced polymer–TTX formulation containing a chemical osmotic enhancer (sodium octylsulfate) into the sciatic nerve of rats, a reliable sciatic analgesic effect was produced for up to 22 days. Liu et al. [89] encapsulated TTX in hollow silica nanoparticles and injected it into the sciatic nerves of rats. The results showed that encapsulated TTX improved nerve block efficiency, prolonged block duration, reduced systemic toxicity, and showed effective targeting compared with free TTX, and the detailed conclusions of this study are shown in Figure 3.

TTX is a highly toxic substance, and thus, it is crucial that we clarify the safe dose of TTX in clinical applications. There have been studies on the systemic toxic effects of TTX in humans and the medicinal safety of TTX, and currently, based on the results of clinical trials and animal models, it has been shown that the oral administration of TTX is nearly 50 times safer than intraperitoneal injection. Kavoosi et al. [90] conducted a randomized, double-blind, controlled clinical study to evaluate the safety and human tolerability of subcutaneous injections of 15, 30, and 45 μg of TTX over three cycles (each 7 days apart) in 25 healthy adults. The results showed that these doses of TTX were safe and tolerable in humans. However, eight subjects in the study experienced multiple minor adverse reactions (abnormal sensations, abnormal oral sensations, headache, dizziness, nausea, and myalgia). Considering that patients may have different physical conditions when they are ill compared to healthy subjects, further studies are required to determine the optimal TTX doses for different patient groups.

## 5. TTX Biosynthesis Mechanisms

**Figure 4:**
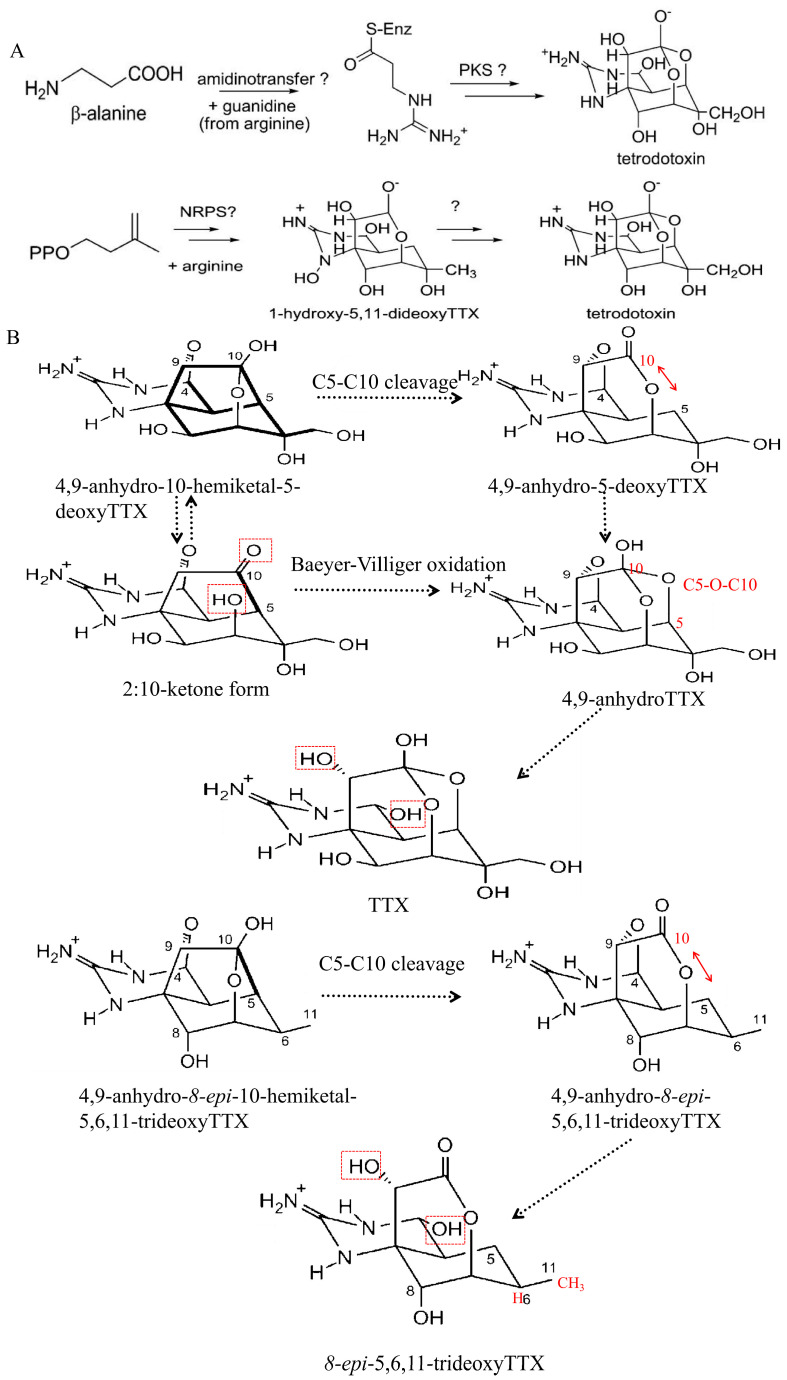

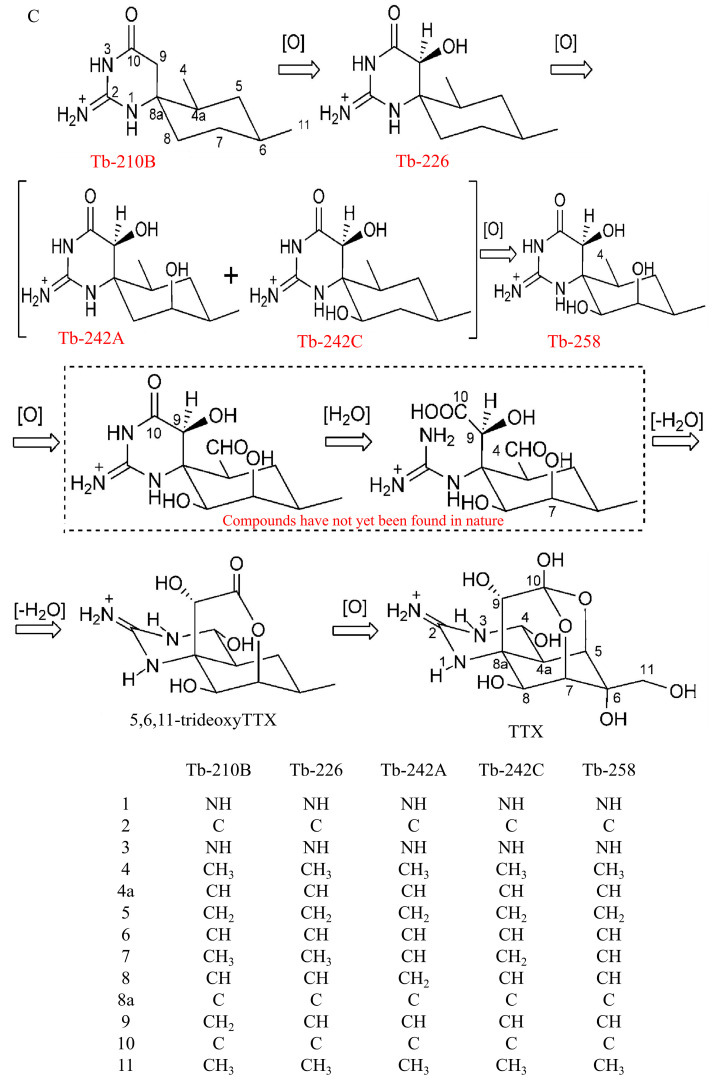
Tetrodotoxin (TTX) biosynthesis processes [93,94,95]. (**A**): arginine synthesis; (1, the synthetic process of polyketone derivatives; 2, the sugar-derived TTX carbon backbone process); “?” represents an as yet unspecified step in the biosynthesis of TTX; (**B**): monoterpene compound synthesis process (labeled red functional group changes position for TTX biosynthesis); The parts marked in red refer to changes in functional groups;C5-O-C10: An ether bond is formed between the fifth and tenth carbons; and (**C**): intermediate compounds synthesis (Tb-210B, Tb-226, Tb-242A, Tb-242C, Tb-258), which are isolated intermediate in the biosynthesis of pufferfish TTX. These figures are re-published with permission from copyright (2018) Chemistry-A European Journal, copyright (2014) Angewandte Chemie International Edition, and copyright (2019) American Chemical Society.

The TTX biosynthetic pathways and related genes have not yet been elucidated. Previous research into TTX-producing strains and marine organisms carrying TTX has resulted in the generally accepted idea that TTX is primarily produced by bacteria and enriched in marine organisms, such as pufferfish species, octopus species, and crab species, through the food chain. Although scientists have attempted to trace the biosynthetic process of TTX using isotope-labeled compounds, these efforts have not yet revealed the key genes involved in TTX biosynthesis. In addition, it is difficult to generate steady cultures of the TTX-producing strains under laboratory conditions, and the amount of toxins they produce rapidly decays. These factors all add to the complexity of the TTX biosynthetic pathway [91]. Recent advances in our understanding of TTX biosynthesis are reviewed, and the synthesis process is shown in Figure 4.

### 5.1. Hypothesis of the Arginine Synthesis Pathway

The molecular backbone of TTX is a 2,4-dioxadamantane ring containing five hydroxyl groups to which a guanidinium group is attached. The guanidinium group of TTX is a key site for binding to the sodium channel and is essential for its toxin activity. Two pathways for the synthesis of TTX from arginine have been identified. First, the process of polyketide derivative synthesis involves arginine that transfers the guanidinium group to the three-carbon unit substrate of TTX biosynthesis via amidinotransferase, followed by the extension of malonyl coenzyme A-derived acetate to form the main chain of TTX [92]. However, how the newly synthesized polyketide carbon backbone is converted to the TTX-specific cage-like structure is unclear, and relevant studies are lacking. Second, the sugar-derived TTX carbon backbone process involves arginine that can be condensed with branched-chain apiose sugar or isoprene units by non-ribosomal peptide synthase (NRPS) to form the general structure of TTX [93]. However, this pathway requires further validation.

### 5.2. Hypothesized Biosynthetic Pathways for Monoterpenes

In 2014, Kudo et al. [12] extracted two TTX analogs from newts, 4,9-anhydro-10-hemiketal-5-deoxyTTX and 4,9-anhydro-8-epi-10-hemiketal-5,6,11-trideoxyTTX, of which 4,9-anhydro-10-hemiketal-5-deoxyTTX was highly correlated with the TTX concentration (Spearman’s correlation coefficient value = 0.88) and was hypothesized to be a biosynthetic precursor of TTX. It was also proposed that the C5-C10 bond in 4,9-anhydro-10-hemiketal-5-deoxyTTX could be oxidized to form 4,9-anhydro-TTX, which can be further hydrolyzed to TTX. Since the carbon skeleton of 4,9-anhydro-10-hemiketal-5-deoxyTTX may be derived from monoterpenes, it was hypothesized that TTX biosynthesis originates from monoterpenes. Furthermore, the presence of multiple cycloguanidine compounds in poisonous newts further substantiates the monoterpene biosynthesis hypothesis. In 2016, Kudo et al. [94] found five cycloguanidinium-based compounds in toxic newts, which further supports the idea that TTX in newts is derived from monoterpenes. In 2019 and 2020, Kudo et al. [12,95] discovered TTX analogs in the newts *Cynops ensicauda pope*, *Cynops*, and *Taricha* that had not been previously identified in marine organisms: 1-hydroxy-8-epiTTX, 1-hydroxy-8-epi-5,11-dideoxyTTX, and TTX biosynthesis shunt compounds (Cep-226A, Cep-228A). Through the N-OH reduction reaction, 1-hydroxy-8-epi-5,11-dideoxyTTX was converted into the intermediate 8-epi-5,11-dideoxyTTX, and this intermediate was verified to be present in two newts, *Cynops* and *Taricha*, which further enriches the hypothesis that terrestrial TTX is derived from monoterpenes.

### 5.3. Hypothesized Biosynthetic Pathways for TTX Analogs

The TTX analogs that were identified in newts have not yet been detected in marine organisms. It is hypothesized that the TTX biosynthetic pathway is different in marine and terrestrial organisms. Ueyama et al. [96] suggested a possible biosynthetic pathway by studying various analogs in the pufferfish *Tetraodon biocellatus*, i.e., the synthesis of 5,6,11-trideoxyttx (the major analog of ttx in marine organisms) via newly discovered TTX-related compounds and, thus, the subsequent synthesis of TTX. This synthetic pathway is very complex and involves oxidation reactions, the hydrolysis of cyclic amides to generate carboxylic acid and acyclic guanidine functional groups, guanidinamide cyclic structures, and the synthesis of 5,6,11-trideoxyTTX lactones. Recent studies have shown that the oxidation of 5,6,11-trideoxy to TTX is further enriched by the discovery of 5,11-dideoxy TTX in the ovaries of the pufferfish *Takifugu poecilonotus* and in the pharynx of the flatworm (*Planocerid* sp. 1). The data show that 5,6,11-trideoxy TTX is first oxidized to 5,11-dideoxy TTX, whereas 5,11-dideoxy TTX is oxidized to 5-deoxy TTX and 11-deoxy TTX, and finally to TTX [97].

The identified biosynthetic pathways involve a variety of key enzymes, such as amidinotransferases, non-ribosomal peptide synthases, polyketide synthases, and Baeyer–Villiger mono oxygenase. These enzymes are also involved in the biosynthetic processes of other toxins, such as saxitoxins (STXs). STXs [68] are small-molecule alkaloids similar to TTX that are neurotoxic. Together with 57 analogs, they are classified as paralytic shellfish toxins. STX biosynthesis in cyanobacteria utilizes an amidinotransferase encoded by the *sxtG* gene. This enzyme transfers the amidino group from arginine to 4-amino-3-oxoguanidinoheptane [98]. Non-ribosomal peptide synthases and polymerized ketolases are multifunctional enzyme complexes that sequentially assemble amino acid and carboxylate-derived precursor building blocks into the product in an assembly-line-like manner. López-Legentil et al. [99] isolated a cluster of non-ribosomal peptide synthetase and polymerized ketolase (NRPS-PKS) hybrid genes associated with brevetoxins synthesis from toxic dinoflagellate *Karenia brevis*. These enzymes are not only involved in the biosynthesis of TTX but also in the synthesis of other toxins. In the future, the screening and identification of genes involved in TTX biosynthesis enzymes to similar gene sequences will be one of the methods explored to elucidate its biosynthetic mechanism.

## 6. Transport and Accumulation of Tetrodotoxin in Organisms

TTX was first discovered in pufferfish in 1909 and has since been identified in a wide range of animals, including aquatic organisms such as crabs, starfish, octopuses, and gastropods [100,101], and amphibians such as newts, frogs, and water lizards [102,103]. Given the wide distribution of TTX in the animal kingdom and the unlikelihood that organisms from different phyla share the same TTX-encoding genes, this further supports the hypothesis that animals are enriched for TTX through the food chain. This is of interest to researchers who aim to elucidate the mechanisms of TTX transport and accumulation in organisms.

For example, pufferfish use TTX as a defense mechanism against predators. When faced with a threat, a pufferfish can expand its body into a spherical shape to deter predators and release TTX from the glands and secretory cells in its skin to repel predators. Female pufferfish can transfer TTX to their eggs to enhance their defense capabilities against predators. Studies have shown that other non-toxic fishes and pufferfishes can recognize TTX by taste and smell, and when common fish inadvertently swallow pufferfish eggs containing TTX, they rapidly spit them out [104]. TTX also functions as an information hormone for pufferfish and is used by females to attract males during the breeding season [105]. In summary, TTX is essential for pufferfish survival. Studies on the dietary habits of pufferfish have revealed that toxic pufferfishes (TTX concentration ≥ 2.2 μg/kg) prefer to consume TTX-containing foods to accumulate TTX [9,106]. Itoi et al. [107] found toxic *Takifugu pardalis* eggs in the gut of *Takifugu niphobles*. The above studies confirm the hypothesis that pufferfish accumulate TTX through the food chain. However, the transport, accumulation, and metabolism mechanisms for TTX in pufferfish have not yet been clearly elucidated. In this section, we summarize our current understanding of the transport and accumulation of TTX in pufferfish.

The concentration of TTX and its accumulation in pufferfish varies significantly between species. Generally, in most species of pufferfish, the highest TTX concentrations are found in the liver, ovaries, and skin [108,109], and the muscle and testis may be non-toxic or have smaller amounts of TTX [34]. The skin is one of the primary TTX-rich tissues, and in 1986, Kodama et al. [110] identified specific exocrine glands or gland-like structures in the skin of several species of pufferfish of the genus *T. poecilonotus* (*T. poecilonotus*, *T. niphobles* and *T. vermiculare radiatum*) and detected high concentrations of TTX in the glands. However, in 2002, Tanu et al. [111] stained skin tissue sections of the pufferfish *Tetraodon steindachneri* using a TTX-specific monoclonal antibody and found that TTX was primarily distributed in the basal and succiform cells; the latter secreted the toxin externally, and no other TTX-secreting glands were identified. It was hypothesized that the skin tissues of different pufferfish species had varied compositions but that they all secreted TTX externally when stimulated. An in vitro study on TTX uptake in skin tissue slices of non-toxic pufferfish by Gao et al. [112] showed that TTX was first taken up by the connective tissue of the skin and then transferred to the basal cells. Mahmud et al. [113] identified TTX in the lysosomes of basal cells from the skin of the pufferfish *Tetraodon nigroviridis*. It was hypothesized that the TTX-binding proteins in the blood of the pufferfish enter the basal cells by diffusion and are then taken up by the lysosomes. There is no clarity as to the manner in which TTX enters the skin connective tissue/succiform cells, whether it is through physical diffusion or a specific uptake mechanism. In 2021, Feng et al. [114] identified a variety of genes and proteins related to TTX transporter accumulation in the skin of the pufferfish *Takifugu flavidus* through multi-omics analysis, reflecting the complexity of the TTX transporter accumulation mechanism: the genes included the *SLC26* family, Fos proto-oncogene (*fos*), Jun proto-oncogene (*jun*), and f immediate–early response genes (*ieg*); the proteins included myosin light chain 2, myosin light chain 4 (MYL4), myosin heavy chain 7 (MYH7), titin (TTN), tropomyosin 2, and tropomyosin 3, which indicated that cardiac muscle contraction and adrenergic signaling in the cardiomyocyte signaling pathway play important roles in TTX accumulation. In addition, TTX accumulation in pufferfish also showed tail age variability. In the TTX administration experiments of non-toxic pufferfish at maturity, TTX in juvenile fish was primarily transported to the skin and then excreted, whereas in adult fish, it was primarily transported to the liver, where it accumulated, and the TTX levels of 15-month-old pufferfish were almost five times higher than those of 6-month-old pufferfish [115,116]. These results indicate that the transport and accumulation of TTX in pufferfish are related to liver development.

**Figure 5:**
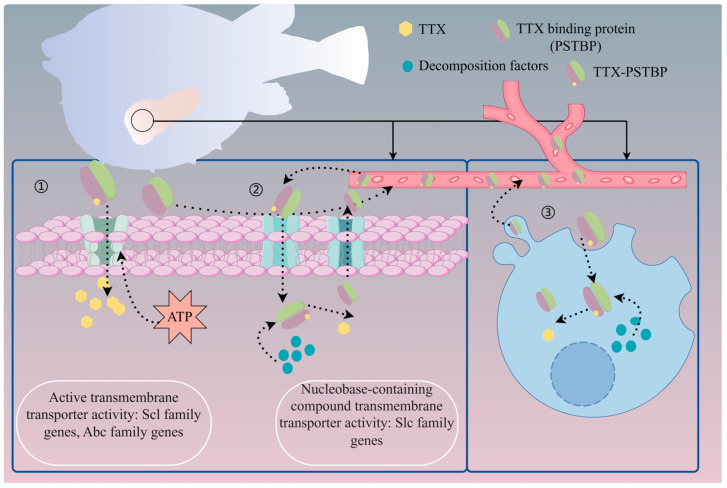
Hypothetical transporter accumulation pathway of TTX in pufferfish liver [117,119,125]. Solid line: physiological processes occurring in the liver of the puffer fish; dashed line: indicates that this process is currently hypothesized and has not yet been confirmed by sufficient data. ➀ Hepatocytes take up only TTX and consume energy through membrane proteins, and the binding protein PSTBP does not enter the cell; ➁ the TTX-PSTBP conjugate enters the hepatocyte through the alkaline compounds transferring the membrane proteins and is then dissociated by acidic catabolic factors into free PSTBP and TTX, and PSTBP then diffuses to the extracellular space through the correlated protein channel; ➂ TTX-PSTBP enters the hepatocyte through cytosolization into hepatocytes, then dissociates into free PSTBP and TTX via acidic catabolic factors, and PSTBP is secreted extracellularly via the cytosol or exosomes. The figure was drawn using figdraw 2.0.

The pufferfish liver is an important transit organ for the transport and accumulation of TTX. Whether TTX is absorbed from the digestive system or injected intramuscularly, TTX accumulates in the liver first and is then transported to the skin, ovaries, and other organs [116,117]. In the liver, TTX is first taken up by pancreatic exocrine cells and then diffuses to hepatic parenchymal cells, and the specific accumulation mechanism is shown in Figure 5. Tatsuno et al. [118] investigated the rate of hepatic uptake using the intramuscular injection of low, medium, and high (30, 100, and 300 μg/each) doses of TTX. The results showed that the liver could take up a fixed proportion of the circulating TTX at a constant rate regardless of the administered concentration. In addition, a TTX- and STX-binding protein, PSTBP, was identified in pufferfish plasma [118,119] and may transport TTX to the liver and other organs via the bloodstream. In addition to PSTBP, Qiao et al. [120] identified a novel TTX-binding protein, peroxiredoxin-1, in the pufferfish *Takifugu bimaculatus*, which was found to promote cellular uptake of TTX.

A number of genes related to TTX transport and accumulation have also been identified. wap65-like, serotransferrin-like, and complement C3-like are genes related to the accumulation of TTX in the livers of adult *T. rubripes* [121]. Kiriake et al. [122] conducted a study on the differential genes involved in the hepatic uptake and accumulation of TTX in juvenile and adult pufferfish *T. rubripes* and showed that there was no difference in the ability of the two species to uptake TTX, but there were differences in the genes associated with hepatic TTX accumulation (Chymotrypsin-like elastase family member 2A-like in the juvenile and Elastase family member 2A-like in the adult). Pufferfish saxitoxin and tetrodotoxin-binding protein-related gene 1 (*PSTBPTr1*) was also identified; Tr1 [123] is only expressed in the liver, and its product is a 120 kDa plasma protein. The Tr1 protein shares 90% similarity with the C-terminal structural domain of PSTBP1, indicating that it may be involved in the delivery and recognition of TTX in pufferfish. The fibrinogen-like protein (flp) genes, *flp-1*, *flp-2*, and *flp-3*, found in the livers of wild pufferfish *Takifugu chrysops* and *Takifugu niphobles*, were linearly correlated with the TTX level and are potential genes related to TTX transport accumulation [124]. Zhang et al. [125] used the transcriptome analysis of differential genes in the livers of toxic and non-toxic pufferfish *T. rubripes*, and the results showed that there were 2262 differential genes between the two and many differential genes participated in endoplasmic reticulum-related pathways and molecular functions (e.g., protein processing pathways in the endoplasmic reticulum, protein export, and metabolism pathways). Because it is now known that the PSTBPs are synthesized in the liver, it was hypothesized that these genes may be involved in the production of TTX carrier proteins and pro-TTX accumulation factors. In addition to the above, Gao et al. [126] conducted toxin administration experiments on the marine species *T. pardalis* and the freshwater species *P. suvattii* and found that different species of pufferfish exhibited the selective accumulation of TTX and paralytic shellfish toxins. Specifically, *T. pardalis*, which naturally carries TTX, tended to accumulate TTX, whereas *P. suvattii*, which naturally carries paralytic shellfish toxins, tended to accumulate paralytic shellfish toxins, which reaffirmed the existence of a complex mechanism for TTX uptake by pufferfish in the liver and other organs.

In toxic female pufferfish, the ovaries are the organ with the highest TTX content; however, the accumulation mechanism has not yet been fully elucidated. The study described found that the level of TTX in the ovary is influenced by a variety of factors, such as the tail age, seasonal changes, and before and after ovulation [9,127]. Ikeda et al. [128] studied the wild pufferfish *Takifugu poecilonotus* and found that TTX in the ovary increased with the gonadal index. Itoi et al. [129] analyzed seasonal changes in TTX levels in the pufferfish *Takifugu niphobles* and showed that both males and females exhibited significantly higher TTX levels during the maturation period (April) and the spawning period (May–July) than during the other periods; furthermore, there was a significant difference in the primary organ of TTX accumulation between males and females during the spawning period in the ovaries for females and livers for males. The localization of TTX in the ovary was clarified first to help elucidate the mechanism of TTX transporter accumulation in the ovaries of pufferfish. In pufferfish *Takifugu vermicularis*, for example, when the ovary was in the late nucleolus perinuclear and yolk granule I stages, TTX was predominantly identified in the nucleus of the oocyte and yolk vesicles, whereas in the yolk granule II stage, TTX was present only in the nucleus of the oocyte [130]. Second, the source of TTX in the ovary was considered; it is currently believed that female pufferfish are influenced by gonadal indices and other factors during maturation, and that TTX is transported from the liver to the ovary and accumulates via the carrier protein PSTBP [130]. However, the localization of PSTBP in the ovary revealed that it was present in the ovarian wall and yolk envelope of the oocyte, which indicates that PSTBP cannot enter the oocyte. In addition to PSTBP, Yin et al. [131] extracted two new TTX-binding proteins, TPOBP-10 and TPOBP-15, from the ovaries of *T. pardalis* and identified TPOBP-10 as a sub-structural domain of yolk proteins and the vWF D-type structural domain as a homologue of yolk proteins. Compared with the vWF D-type structural domain of TTX-free fish, TPOBP-10 was found to be substituted with acidic amino acid residues (Asp and Glu), basic amino acid residues (Lys), and polar amino acid residues with hydroxyl groups (Ser), which were hypothesized to be the binding sites of TTX-binding proteins. Qiao et al. [17] identified the TTX-binding protein von Willebrand factor type D (VWD) in the yolk proteins of the pufferfish *Takifugu flavidus* and elaborated on the key amino acids for TTX binding (Val, Asp, and Lys). The discovery of TTX-binding proteins in the ovary has helped to elucidate the mechanism of TTX transport and accumulation in the ovary. However, studies on the mechanism of TTX accumulation and related genes or proteins and on the mechanism of TTX transport and accumulation within tissues and between organs are still limited and further research is required.

## 7. Tetrodotoxin Detection Methods

To reduce the risk of TTX poisoning due to edible aquatic products, the application and development of simple and sensitive TTX detection methods have become a major focus of scientific research. The methods currently used for TTX detection are mainly categorized into bioassays, immunoassays, and instrumental analyses [132]. Biological assays include the mouse bioassay (MBA), and cell bioassay (CBA); immunoassays include the enzyme-linked immunoassay (ELISA), and lateral chromatography assay (LFA); and instrumental analyses include the liquid chromatography–fluorescence detector (LC-FLD), gas chromatography–tandem mass spectrometry (GC-MS), liquid chromatography–mass spectrometry (LC/MS), liquid chromatography–tandem mass spectrometry (LC-MS/MS), high-performance liquid chromatography–tandem mass spectrometry (HPLC-MS/MS), and ultra-performance liquid chromatography–tandem mass spectrometry (UPLC-MS/MS). These TTX detection methods are summarized in Table 2.

Currently, LC/MS and ELISA are commonly used for the detection of TTX in edible aquatic organisms. LC/MS, LC-MS/MS, HPLC-MS/MS, and UPLC-MS/MS have the advantages of rapid analysis, high detection accuracy, and high sensitivity; however, the complexity of the sample pre-processing and the use of expensive equipment has limited its large-scale sample detection. Compared with instrumental analyses, ELISA and LFA are more economical detection methods, which do not require expensive detection instruments, are simple to operate, and are suitable for the initial screening of large-volume samples. With the pursuit of simpler and more sensitive TTX detection methods, the emerging biosensors for TTX detection have received a lot of attention from scholars in recent years, and the following summarizes the development of biosensors for TTX detection in recent years.

Biosensors utilize biomolecules as recognition elements, with cells, antibodies, and aptamers being the commonly used biological recognition elements in TTX sensing. TTX biosensors can be classified into three main types: cellular biosensors, immunosensors, and aptamer sensors [146]. Cellular biosensors mainly use neuronal cells and cardiomyocytes as recognition elements, which produce different signal changes upon sensing a stimulus, and the sensors convert these changes into measurable electrical signals. Alkassar et al. [147] showed that the Neuro-2a cellular biosensor was able to detect standardized solutions of ciguatoxin and TTX, as well as the toxicity of samples from toxin-carrying fish (*Seriola dumerili* and *Lagocephalus sceleratus*). Campàs et al. [148] used a Neuro-2a cell-based automated patch clamp system to detect TTX levels in pufferfish samples with an LOD of 0.05 mg TTX equiv/kg.

Aptamer sensors utilize TTX-specific binding aptamers as recognition elements. An aptamer is a piece of DNA or RNA that can selectively interact with a specific target because of its strong affinity. Compared with antibodies, aptamers have significant advantages, including the ease of in vitro screening, ease of preparation, high specificity, ease of replication during chemical synthesis, low batch-to-batch variation, ease of modification, and stability for long-term storage. Consequently, aptamers show great potential in a variety of applications. Aptamers with strong affinity for targets are generally obtained using the standard process of SELEX (Systematic Evolution of Ligands by Exponential Enrichment) [149]. Li et al. [150] identified two thermally stable aptamers (Tv-51 and AI-57) with high affinity for TTX and aptamer variants (Tv-46 and AI-52) with higher affinity and TTX specificity through SELEX studies. Lan et al. [151] constructed a fluorescent aptamer sensor for TTX detection using berberine as the signaling factor, the TTX-aptamer as the detection unit, and nucleic acid exonuclease I (Exo I) as an eliminator to eliminate the background fluorescence of TTX; it was shown that the detection results were stable and the detection limit of TTX was 11.0 pM, which reflected the high sensitivity and specificity of the TTX–aptamer sensor. Zhang et al. [152] used a magnetic bead-aptamper and triple-cycle amplification techniques to detect TTX, and the results showed that the detection limit was 0.265 pg/mL, and the sensitivity was three times higher than with the Elisa kit, with reliable sensitivity, specificity, and stability.

Immunosensors utilize specific immune responses between antibodies and antigens. In contrast to earlier single-signal immunosensors, recently, there have been a series of dual-mode sensing platforms created using two or more response mechanisms with relatively independent signal transduction, especially electrochemical/colorimetric mechanisms. Li et al. [153] constructed a non-toxic electrochemical/colorimetric dual-mode sensor using a high-affinity antibody to TTX (anti-TTX mAb) and synthesized a biomimetic mineralized material that encapsulates both the anti-TTX mAb and horseradish peroxidase (HRP). The electrochemical/colorimetric dual-mode immunosensors optimized by this study are well-suited for the detection and monitoring of trace levels of toxins. Li et al. [139] investigated a lateral flow immunosensor based on a gold nanoparticle-labeled monoclonal antibody probe, which allowed for the affinity reaction of an antigen. This immunosensor assay can be completed in 10 min with an LOD = 10 μg/kg. In addition, nanoscale metal ions applied to the SPR (surface plasmon resonance) immunosensor enhance the detection signal. Yao et al. [154] developed a highly sensitive and interference-resistant dual-mode aptasensor for the detection of TTX from 100 pg/mL to 10 μg/mL by exploiting the electrochemical and surface-enhanced Raman scattering activity of Ag@Cu_2_O NPs. In addition, fluorescent sensors combining nanoscale metal–organic frameworks (MOFs) and aptamers also show great potential.

Although biosensors show great potential for the detection of TTX, there are some challenges currently limiting their development, such as possible spatial site-blocking effects during immobilization, the non-specific adsorption of beads, possible conformational changes in immobilized target molecules, the complexity of the experimental process, the problems of the immobilized target molecules, and the complexity of the experimental process [155]. Hybrid detection methods can be used to improve the sensitivity, accuracy, and specificity of the assay. Antibody and aptamer sandwich methods have reportedly been used to detect TTX. These methods include the mixture of the D3 aptamer and monoclonal antibody, which is not only highly sensitive (detection limit of 310 pg/mL, equivalent to 970 pM) but also highly specific, and the results of the assay are not affected by other marine toxins [156]. Nanoscale metal–organic frameworks (MOFs) show great potential for sensor applications, and MOFs are characterized by low cost, high productivity, and performance. Dou et al. [157] studied NMOFs-Aptasensor 1 and NMOFs-Aptasensor 2 with detection limits of 1.17 nM and 3.07 nM for STX and TTX, respectively, and these sensors, which have remarkable stability, pH independence, and good selectivity, have now been used to detect toxins in shellfish samples.

## 8. Clinical Manifestations and Treatment of TTX Poisoning in Humans

TTX poisoning incidents occur mainly in Japan, Taiwan, China, and Southeast Asia. Due to its high toxicity, TTX can lead to acute poisoning. Clinical symptoms may include perioral tingling, ocular paralysis, muscle weakness, nausea, vomiting, a floating sensation, impaired consciousness, respiratory distress, vasodilatory shock, and cardiac arrhythmias. The development of these symptoms has been associated with a variety of factors, including (1) the amount and part of the toxic pufferfish consumed; for example, in the 2008 Bangladesh outbreak, the poisoned patients who died as a result of consuming TTX-carrying pufferfishes all consumed more than 100 g [20]. (2) Patients with underlying health issues are also at high risk; in the incident of the accidental ingestion of wild moon-tailed pufferfish (*Lagocephalus lunaris*) by fishermen in Taiwan, China, in 2001, the patient who died had consumed a small portion but died because of cardiac bradycardia and multi-organ failure due to the synergistic effect of diabetic neuropathy and the sodium channel blocking property of TTX [158]. A 52-year-old woman suffering from uremia after consuming pufferfish soup, and 4 h later, she experienced perilabial numbness, dizziness, unsteadiness, and difficulty in swallowing, but her husband, who ate at the same time as her, did not show any symptoms of poisoning [159]. Therefore, the physical health status of the poisoned patient affects the severity of the TTX poisoning symptoms. (3) Timely medical treatment is provided after poisoning. The onset time of TTX poisoning varies greatly among individuals, and according to the relevant cases, the symptoms predominantly start after 5–45 min [160]. However, there are documented cases in which the symptoms start approximately 2–3 h after ingestion [161].

Due to the lack of specific antidotes for TTX, patients with poisoning are usually treated with emetics and gastric lavage or other auxiliary treatments to promote the elimination of TTX from the body and to reduce the symptoms of poisoning. Patients with mild TTX poisoning and those with non-life-threatening poisoning generally recover within 24 h [66]. The rest of the patients with non-life-threatening TTX poisoning usually recover within 5 days after prompt emesis or other auxiliary treatments [162]. In clinical practice, the symptoms of TTX poisoning usually subside within 5 days after prompt emetic or other auxiliary treatment. In clinical practice, adjunctive therapeutic measures are usually taken according to the patient’s symptoms: gastric lavage for patients who have not yet vomited naturally and the use of activated charcoal within 1 h of ingesting TTX; if the patient has motor paralysis, cholinesterase inhibitors (neostigmine bromide) can be given to inhibit the breakdown of acetylcholine at the neuromuscular junction [163]. In patients with symptoms such as respiratory failure or hypotension, mechanical ventilation and vasopressor medications (noradrenaline, d-amphetamine, and phenylephrine) are administered [164]. In patients who ingest high doses of TTX, hemodialysis may be attempted. Since TTX is a small molecule and does not bind to proteins in the blood, dialysis partially removes free TTX from the blood vessels and reduces the possibility of death. There are clinical cases where critically ill patients receiving continuous venovenous hemofiltration and intermittent dialysis showed significant symptomatic improvement 15 h after starting dialysis [165,166]. Hemodialysis is also a necessary treatment route for uremic patients with TTX poisoning. A Japanese uremic patient with TTX poisoning continued to experience unrelieved symptoms after TTX poisoning due to the accumulation of TTX in the body because of renal function problems, but his symptoms improved significantly after undergoing hemodialysis [167]. However, some scholars suggest that the water solubility of TTX is minimal, that the effect of hemodialysis is not significant, and that healthy individuals with good renal function can eliminate TTX through their own metabolism when the symptoms of intoxication are not serious without the assistance of hemodialysis.

## 9. Conclusions and Future Directions

This review discusses the origin, analogs, medicinal value, detection methods, biosynthetic processes, and mechanisms of the transport and accumulation of TTX in pufferfish. Despite significant research advancements regarding TTX and its analogs in recent years, further investigation in this field is still necessary. To date, studies on TTX have primarily focused on four key areas. The first area is the prevention and reduction of TTX and analog poisoning incidents. TTX and its analogs have been identified in numerous species, particularly in edible aquatic organisms. Research aimed at developing rapid, sensitive, and user-friendly detection methods, as well as elucidating the transport and accumulation mechanisms of TTX and its analogs in edible organisms, is of great importance. The data obtained from such studies can help individuals avoid poisoning from TTX and its analogs. The second area of focus has been on scaling up the production of TTX and its analogs. Currently, TTX and its analogs are primarily obtained via isolation from the tissues of TTX-carrying organisms. However, this strategy requires a substantial number of raw materials, and yields are often low. In addition, although the chemical synthesis of TTX has been reported, mass production remains challenging due to cumbersome and time-consuming procedures, high costs, and low yields. Therefore, the biosynthetic processes involved in the production of TTX and its analogs need to be further elucidated to develop low-cost and sustainable supply routes for TTX. The third area of focus has been to elucidate the mechanism by which the TTX transporter accumulates in organisms, clarifying the regulatory relationship between its genes and proteins and providing a theoretical basis for the mechanism of TTX enrichment in these organisms. The fourth area of focus has been on the medicinal value and use of TTX as a sodium ion blocker, which exhibits significant local anesthesia and analgesic effects without the addictive side effects associated with opioids. These characteristics suggest that TTX has substantial clinical potential. The follow-up on its clinical applications, standardization of medication, and regulatory control will be crucial for the future applications of TTX.

## Figures and Tables

**Table 1:** TEF assessment of TTX analogs.

**Analog**	**TEFs**	**Reference**
TTX	1	[52,53,54,55,56,57,58,59]
4-epiTTX	0.156	
6-epiTTX	0.166	
11-deoxyTTX	0.140/0.107	
6,11-dideoxyTTX	0.023/0.035	
11-oxoTTX	0.75	
11-norTTX-6(R)-ol	0.17	
11-norTTX-6(S)-ol	0.185/0.404/0.238	
5-deoxyTTX	0.01	
6-deoxyTTX	0.32	
5,11-dideoxyTTX	0.75/0.027	
5,6,11-trideoxyTTX	0.011/0.001	
4,4a-anhydroTTX	0.0014	
4,9-anhydroTTX	0.02	

**Table 2:** Summary of TTX detection methods.

Methods	Advantages	Limitations	Applications
MBA [5,133,134]	Experimental phenomena are intuitive and provide true information about the toxicology of TTX without the expensive instrumentation.	Individual differences in mice, poor accuracy, and reproducibility of test results; there are ethical issues and the high cost of experimentation.	Japanese authorities define 1 MU as the dose of TTX that induces death in 20 g ddY male mice within 30 min, and 1 MU is equivalent to 0.22 μg TTX [5].
CBA	There are no ethical issues, and its operation is relatively simple and more sensitive than MBA.	Requires a long cell culture time, highly demanding experimental conditions, and is greatly influenced by subjective judgment.	Gallacher and Birkbeck [135] used this method to measure samples with a limit of detection (LOD) of 20 ng/mL.
ElISA [136,137]	No need for complex and expensive testing instruments. Simple operation method with high sensitivity.	Conventional ELISA has relatively low sensitivity, and antibody preparation is cumbersome and costly. The stability and reproducibility of antibodies prepared in different batches may vary.	Vlasenko et al. [138] developed a competitive ELISA using polyclonal antibodies. The LOD and limit of quantification (LOQ) for TTX were 25.54 and 777.34 ng/mL, respectively. The detection range was 25.54–2500 ng/mL.
LFA [139]	Simple and fast, with no need for complicated and expensive testing instruments.	Semi-quantitative analysis based on visual judgment.	The visual detection of TTX and OA was accomplished within 10 min with limits of detection (LOD) of 15 and 0.75 ng/mL, respectively [140].
LC-FLD [38]	The earliest instrumental method for the quantitative determination of TTX.	This method requires specialized equipment and cumbersome operation, is susceptible to the influence of fluorescent impurities, and has high requirements for samples.	
GC-MS	Rapid detection and quantitative determination of TTX.	TTX needs to be derivatized into substances with volatile properties, and TTX analogs cannot be distinguished.	Man et al. [141] used GC-MS to detect TTX in river herring samples and determined the LOD to be 0.5 μg/g with a detection time of 8.2 min.
LC-MS	The separation and characterization capabilities of this method are efficient, rapid, sensitive, and accurate, enabling high throughput and trace analysis.	The large volume of samples required, the complexity of pre-treatment, the use of expensive equipment, and the high cost of detection limit this method’s application in large-scale sample detection.	Horie et al. [142] used LC-MS to detect TTX in river herring samples and determined that the LOD = 0.1 mg/kg with recoveries ranging from 77.7 to 80.7%.
LC-MS/MS	The separation and characterization capabilities of this method are efficient, rapid, sensitive and accurate, enabling high throughput and trace analysis.		Knutsen et al. [143] assayed TTX levels in gastropods and bivalves and determined an LOD range of 0.1–25 μg/kg.
HPLC-MS/MS	Three times lower LOD for HILIC-MS/MS compared to LC-MS.		The HPLC-MS/MS method established by Ye et al. [144] has a wide detection range (0.2–100 ng/g), low detection limit (0.2 ng/g), and high accuracy (recoveries of 90.5–107.2%).
UPLC-MS/MS	UPLC-MS/MS offers shorter separation times, higher separation efficiencies, and better separations than LC-MS.		Meng et al. [145] coupled solid-phase microextraction with UPLC-MS/MS for the determination of TTX in dark porpoise and puffer fish (Puffer gourami). The LOD and LOQ were 32 and 150 ng/g, respectively.

MBA: mouse bioassay, CBA: cell bioassay, ELISA: enzyme-linked immunoassay, LFA: lateral chromatography assay, LC-FLD: instrumental analyses include: liquid chromatography–fluorescence detector, GC-MS: gas chromatography–tandem mass spectrometry, LC-MS: liquid chromatography–mass spectrometry, LC-MS/MS: liquid chromatography–tandem mass spectrometry, HPLC-MS/MS: high-performance liquid chromatography–tandem mass spectrometry, UPLC-MS/MS: ultra-performance liquid chromatography–tandem mass spectrometry.
